# Just noticeable differences for elbow joint torque feedback

**DOI:** 10.1038/s41598-021-02630-3

**Published:** 2021-12-07

**Authors:** Hubert Kim, Alan T. Asbeck

**Affiliations:** Mechanical Engineering Department, Virginia Polytechnic Institute and State University, Blacksburg, VA 24061 USA

**Keywords:** Musculoskeletal system, Motor control

## Abstract

Joint torque feedback is a new and promising means of kinesthetic feedback imposed by a wearable device. The torque feedback provides the wearer temporal and spatial information during a motion task. Nevertheless, little research has been conducted on quantifying the psychophysical parameters of how well humans can perceive external torques under various joint conditions. This study aims to investigate the just noticeable difference (JND) perceptual ability of the elbow joint to joint torques. The paper focuses on the ability of two primary joint proprioceptors, the Golgi-tendon organ (GTO) and muscle spindle (MS), to detect elbow torques, since touch and pressure sensors were masked. We studied 14 subjects while the arm was isometrically contracted (static condition) and was moving at a constant speed (dynamic condition). In total there were 10 joint conditions investigated, which varied the direction of the arm’s movement and the preload direction as well as torque direction. The JND torques under static conditions ranged from 0.097 Nm with no preload to 0.197 Nm with a preload of 1.28 Nm. The maximum dynamic JND torques were 0.799 Nm and 0.428 Nm, when the arm was flexing and extending at 213 degrees per second, respectively.

## Introduction

Joint torque feedback (JTF) is the sense of being guided or given information by a joint torque generated from a wearable device. This method of haptic feedback is analogous to training someone in how to move via one person manually moving another person’s joint through different trajectories. Typically, this is used in rehabilitation, where someone is re-learning a motor skill, or it can also be used to teach someone a new skill. Joint torque feedback could also be used to convey a general continuous signal to a person, similar to force feedback with a joystick. JTF is a new type of haptic feedback, and has only sparsely been explored^1–4^.

**Figure 1:**
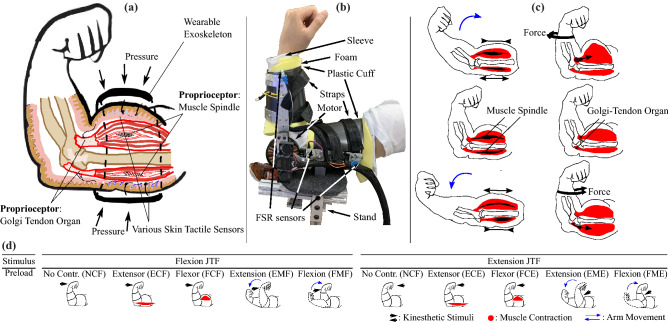
(**a**) Illustration of dissected arm view, showing the primary proprioceptors involved in joint torque sensing as well as how the exoskeleton applies pressure and masks skin receptors. (**b**) Picture of a participant’s arm with the exoskeleton worn, showing the exoskeleton components. (**c**) Illustration of how each propioceptor is triggered under different joint conditions. The left column shows how the Muscle Spindles (MS) lengthen or shorten depending on the arm’s movement. The right column illustrates how isometrically contracted flexor or extensor activity affects the Golgi–Tendon Organ (GTO). (**d**) Illustration of the ten studied torque interactions, showing the kinesthetic stimuli (black arrows), contracted muscles (red highlighted muscle shapes), and direction of arm motion (blue arrows).

In general, haptic information involves mechanotactile sensors, vibrotactile sensors, and proprioception^5–7^. Most prior research has focused on skin receptors due to their ease of stimulation, and has specifically focused on the spatial resolution and sensitivity of skin sensors, such as through vibrotactile^8^, skin stretch^9^, and mechanotactile^10^ feedback. In contrast, this study focuses on the proprioceptors within the arm. Unlike the haptic modalities detected by sensors located under the skin, proprioceptors are situated inside a joint—inside the muscles and tendons^5,6^ (Fig. 1a). The primary afferent joint proprioceptors are Muscle Spindles (MS) and Golgi Tendon Organs (GTO), and they are located in muscle fibers and tendons, respectively^6^ (illustrated in Fig. 1a, c). MS are broadly known to detect the change of length and rate of length change of muscle fibers, and thus detect the position and movement of a joint; GTO are used to measure the tension of the muscle fibers^6^. Our objective is to explore using these sensors to convey haptic information to a person through JTF. This is natural feedback in the sense that these are the same sensory organs—proprioceptors—that we use in daily activities to get feedback about our body’s position and the forces in our muscles. In motion training scenarios, conveying information through the proprioceptors is typically referred to as kinesthetic or proprioceptive feedback^5,6^. Kinesthetic feedback usually consists of position or velocity feedback, or force feedback applied through a handle or joystick. Joint torque feedback is a subset of kinesthetic feedback, where we are specifically concerned with the effects of torques applied across a joint.

Some prior work has been done that is broadly related to the proprioceptive sensors: researchers have quantified how much torque is required to drive a joint passively^11^, and the amount of torque a joint can generate during isometric contraction has also been characterized^12–14^. Also, human perception when the arm is passively-driven has been explored^15^. Early studies utilized a contralateral limb-matching test to investigate the force resolution. Subjects’ perceptual ability was determined based on how well they could reproduce the left arm’s perceived force with the right arm^16^. Researchers also explored identifying force magnitudes presented with a stylus^17^. However, the torque that encourages a person’s voluntary arm motion has not been fully characterized.

For motion training, understanding the minimum detectable torque at a joint is important. In prior work on motion training, researchers have identified two extremes: a fully patient-driven strategy, where no external assistance is provided, and a fully robot-driven strategy where the robot drives a person’s limb through a desired trajectory and the person does not need to exert any effort whatsoever^18,19^. Studies have found that the optimal assistance to maximize learning should be strong enough to cue the person to move and to realize when they have made an error, but not strong enough to hinder the subject’s voluntary motion or fully constrain the wearer’s desired motion^18,20^. To meet these requirements, the torque from a wearable device for motion training should be relatively small but still convey information to the wearer, similar to how vibrotactile motors convey information without causing motion. Thus, it is crucial to understand the minimum perceptual limits of joint torques, in order to optimally convey information through kinesthetic guidance.

Other benefits of understanding the human perceptual resolution include optimizing exoskeletons, teleoperation, and Virtual Reality (VR) systems. For rehabilitation and motion training applications, examining the minimum noticeable level of torque provides an insight into how much parasitic torque is permitted in an exoskeleton or force feedback system without disturbing the wearer. Parasitic torque refers to an unwanted resistance torque when a person moves. It can be induced by several reasons, such as friction, a control loop’s latency, or noise in force feedback sensors. State-of-the-art powered exoskeletons can have parasitic torques of 0.3–1 Nm^21–23^; additionally, Schiele et al. found that the interaction torque between an exoskeleton and wearer due to mechanical constraints and misalignments could be as large as 1.46 Nm^24^.

Understanding the human perceptual resolution is also important in fields where the transmission of physical interaction is required, such as teleoperation or haptic motor learning^25^. The technique called lossy compression utilizes the perceptual resolution for selectively eliminating haptic information outside of the human perception threshold^25^; the reduced data size can improve the efficiency of teleoperation or VR systems.

In this paper, our objective is to understand the minimum necessary amount of external torque for a person to feel its presence (i.e., the Just Noticeable Difference (JND) torque), both when a joint is stationary (static condition) and when it is moving (dynamic condition). The current work is an extension of a prior experiment that solely examined the static condition^3^. In the static condition, a fixed reference torque is applied to the elbow joint and the subject holds stationary to resist that torque, thereby isometrically contracting their arm muscles. On top of the fixed reference torque, additional test torques are applied briefly in order to measure the JND. In the dynamic condition, the arm moves back and forth continuously, and test torques are applied briefly when the arm is at a near-constant velocity. In all of the experimental conditions, pressure and touch sensations from the skin are masked^26^ over the arm area so as to isolate the proprioceptors inside the arm during Joint Torque Feedback.

Exploring the human perception under the joint’s dynamic condition is motivated by our pilot test results^4^ in which a person was following a continuous trajectory while being guided by JTF. We noticed that the arm motion showed a staircase trend: the person felt the JTF at first, and moved their arm. Then, they paused for a moment, since while moving they could not sense the stimulus. The person waited until they could sense the torque, then moved again. This effect suggests that arm speed affects, and masks, the sensation of torque at a joint. The relationship between afferent signals and the masking effect due to movement has been characterized as backward masking^27–29^. Collins et al. applied an electric twitch to the right extensor carpi ulnaris and found that active and passive movement contributed to 37–40% of velocity-dependent attenuation of the muscular sensation with the electric stimulation input^28^. Also, Chapman et al. exerted small electric charges after different delays to the index finger skin before and after the onset of active or passive elbow extension movement^27^. The user response was delayed 38 ms more with active elbow motion as compared to passive motion, indicating that motor commands from the central nervous system contribute to tactile suppression.

Similarly, other researchers conducted perceptual studies of the whole arm with a haptic stylus^1,25,30^. The researchers found that the force feedback magnitude and arm speed influenced the JND, and the observed behavior seemed to have a slope following Weber’s law. In psychophysics, our sensory organs are argued to follow Weber’s law where the ratio of a detectable stimulus change to the initial stimulus intensity is a constant. Correspondingly, this study addresses different JND torques at each joint state (preload torque and joint speed) in terms of Torque Slope (measured JND per preload and measured JND per joint speed).

Several researchers have studied a subset of external conditions affecting the torque perception of the arm. Zadeh et al. studied the JND of kinesthetic stimuli that influenced the whole arm, with a hand-held stylus display^25^ and with the arm in motion. Feyzabadi et al. also used a stylus-based haptic device to study the force perception of the wrist, elbow, and shoulder joints. For the elbow specifically, Feyzabadi et al.’s experiment examined flexional motion ranging from $20^{\circ }$–$80^{\circ }$ with a preload in the flexional direction. Similarly, Thomas et al. employed JTF for replacing the tactile sensation of amputees^2^. Cross-modal matching was conducted between the vibrotactile and the joint torque feedback. The torque was applied in the flexional direction, with the arm moving in free motion. These studies are useful yet do not fully address all of the conditions that would arise during motion training, i.e., different preloads and different speeds with the JTF in various directions. Furthermore, measuring the perception ability of a proximal joint (i.e., the elbow) with a distal manipulator (i.e., a hand-held stylus) could yield different results than observations through direct joint torques. For example, the perceptual resolution at the elbow using a hand-held device^1^ could be affected by the neural receptors in the whole arm’s afferent pathway. Also, these studies with a stylus did not isolate the proprioceptive sensors: there is the chance that skin receptors in the hand holding the stylus could sense forces through pressures, skin stretch, or other feedback signals, giving additional information to the test subject beyond that from the proprioceptors.

**Table 1:** Description of the different conditions examined in this paper.

NTF	Neutral, Flexional torque	Exoskeleton applies a flexion torque while the arm is posed at neutral without muscle contraction or arm movement
ECF	Extensor muscles Contracted, Flexional torque	Exoskeleton generates a flexion torque while the Extensor muscles are isometrically contracted
FCF	Flexor muscles Contracted, Flexional torque	Exoskeleton generates a flexion torque while the Flexor muscles are isometrically contracted
EMF	Extensional Movement, Flexional torque	Exoskeleton generates a flexion torque while the arm is moving in the extensional direction
FMF	Flexional Movement, Flexional torque	Exoskeleton generates a flexion torque while the arm is moving in the flexional direction
NTE	Neutral, Extensional torque	Exoskeleton generates an extension torque while the arm is posed without muscle contraction or arm movement
ECE	Extensor muscles Contracted, Extensional torque	Exoskeleton generates an extension torque while the Extensor muscles are isometrically contracted
FCE	Flexor muscles Contracted, Extensional torque	Exoskeleton generates an extension torque while the Flexor muscles are isometrically contracted
EME	Extensional Movement, Extensional torque	Exoskeleton generates an extension torque while the arm is moving in the extensional direction
FME	Flexional Movement, Extensional torque	Exoskeleton generates an extension torque while the arm is moving in the flexional direction

In this paper, we examine ten different test conditions to completely characterize the arm’s response to both stationary (static) and in-motion (dynamic) conditions (Fig. 1d; Table 1). The test conditions involve arm muscles’ isotonic and isometric contraction representing dynamic and static joint conditions. All of the static experiments were conducted at the arm’s neutral condition ($45^\circ$). All test results were analyzed to derive the participants’ temporal, spatial, and directional perceptions. The static test condition involves 0.89 Nm and 1.28 Nm of preload. In the dynamic condition, the subject’s arm moves at approximately $100^\circ \mathrm {/s}$ and $200^\circ \mathrm {/s}$, which is equivalent to the elbow moving through a range of around $80^{\circ }$ at 0.5 Hz and 1 Hz. In designing the arm’s movement, the highest speed does not exceed 2 Hz based on Neilson et al.^31^.

## Results

### Static JND torques

**Figure 2:**
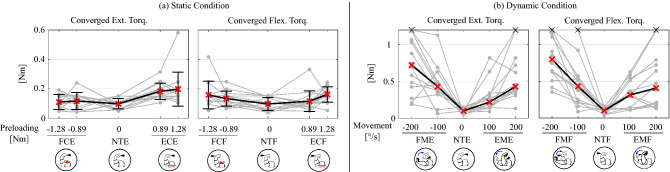
Summary of the converged torque values. For the icons below the x-axis illustrating each experimental condition, please refer to Fig. 1d. (**a**) The JND resulting from the static tests. All subject data is shown with gray lines, and the black line is the mean of the data. (**b**) The JND torque from dynamic tests, where the arm is in motion at different speeds. The black line is the median of the data. The black $\times$s indicate that the data exceeds the maximum device output, which is 1.1 Nm.

The results for the static tests ($N=14$) are shown in Fig. 2a. A summary of the means and standard deviations is listed in Table 2. Each of the conditions in Fig. 2 and Table 1 has two levels of fixed effects, except for the neutral conditions (NCF and NCE). The effect level is followed by the label. For example, ECF has 0.89 Nm (ECF1) and 1.28 Nm (ECF2) of preload torque pulling toward the flexional direction (that leads to the extensor muscles contracting).

**Table 2:** Unpooled means ± standard deviations of the static JND torque and torque slope.

	FC2	FC1	NT	EC1	EC2
Ext. Torq. [Nm]	$0.109 \pm 0.051$	$0.116 \pm 0.058$	$0.098 \pm 0.035$	$0.182 \pm 0.056$	$0.197 \pm 0.116$
	[0.072, 0.146]	[0.080, 0.153]	[0.061, 0.135]	[0.145, 0.219]	[0.160, 0.234]
Ext. Torq. Slope [Nm/Nm]	$0.011 \pm 0.053$		$\cdot$	$0.081 \pm 0.078$	
	[− 0.020, 0.041]			[0.050, 0.111]	
Flx. Torq. [Nm]	$0.158 \pm 0.094$	$0.133 \pm 0.051$	$0.097 \pm 0.045$	$0.116 \pm 0.071$	$0.162 \pm 0.053$
	[0.123, 0.193]	[0.098, 0.168]	[0.061, 0.132]	[0.081, 0.151]	[0.126, 0.197]
Flx. Torq. Slope [Nm/Nm]	$0.047 \pm 0.050$		$\cdot$	$0.046 \pm 0.039$	
	[0.016, 0.077]			[0.015, 0.076]	

The first row (Ext. Torq.) corresponds to the FCE2, FCE1, NTE, ECE1, ECE2 trials, while the third row (Flx. Torq.) corresponds to the FCF2, FCF1, NTF, ECF1, ECF2 trials. (Flx. = flexional; Ext. = extensional; Torq. = torque). The second and fourth rows show the corresponding mean Torque Slopes (i.e., mean of the torque slopes computed for each subject). The second row contains FCE then ECE, and the fourth row contains FCF then ECF. Brackets indicate the 95% confidence intervals [LL, UL].

**Figure 3:**
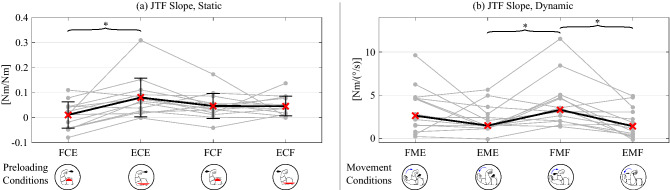
Summary of the subjects’ torque slopes. For the icons below the x-axis illustrating each experimental condition, please refer to Fig. 1d. (**a**) The JND Torque slopes corresponding to static tests. All subject slopes are shown with gray dots, the black line is the mean of the data, and error bars are the standard deviation. The data was analyzed with a Repeated Measures ANOVA followed by a Tukey post-hoc test. FCE and ECE were statistically signficantly different with $p = 0.002$. (**b**) The JND Torque slopes from dynamic tests, where the arm is in motion at different speeds. The black line is the median of the data. The data was analyzed with the Kruskal–Wallis test and followed by the Wilcoxon signed-rank test to compare pairs of conditions. Statistically significant pairs ($p<0.05$) are indicated with asterisks.

The best-fit torque slopes for the static condition (i.e., the slope of the least-squares regression line passing through the Neutral condition and the two levels of torque for a given condition) were calculated for each subject. Figure 3a shows all subjects’ data as grey dots with mean and standard deviation as black lines with error bars; the mean torque slopes are also shown in Table 2. A Repeated Measures ANOVA yielded significant differences between conditions ($F(3,39) = 4.896,p = 0.006$). A Tukey post-hoc test revealed that the FCE and ECE torque slopes were significantly different ($p = 0.002$).

A Kaiser–Meyer–Olkin (KMO) test indicated that factor analysis was not informative (KMO = 0.460) and Bartlett’s test was not significant ($X^{2}(3) = 6.322, p = 0.097$), meaning that there was no strong correlation between each subject’s response across the different conditions. All of the static conditions had response times of $0.445 \pm 0.108$ s, and these were not significantly different from each other per a Repeated Measures ANOVA.

### Dynamic JND torques

During the dynamic condition, subjects moved their arms through a mean $\pm$ Std.Dev. range of $76.4^\circ \pm 7.5^\circ$. At the midpoint of the cycle, the mean absolute arm velocity was $97.1^\circ /$s$\pm 23.2^\circ /$s for the 0.5 Hz conditions and $213.1^\circ /$s $\pm 23.5^\circ /$s for the 1 Hz conditions. The entire cycle took $2.06 \pm 0.01$ s for the 0.5 Hz conditions and $1.04 \pm 0.04$ s for the 1 Hz conditions.

The results for the dynamic JND tests are shown in Fig. 2b, and the medians of the data are given in Table 3. The dynamic measurements involved some trials where the converged JND torque values were beyond the hardware limit (1.1 Nm); these are shown in Fig. 2b with “$\times$” symbols. The arm’s movement speed was a fixed effect. In the dynamic conditions, there were two measured speeds, which were two levels of the fixed effect. For example, the EME has two levels of extensional movement speed: 0.5 Hz (EME1) and 1 Hz (EME2). Note that FMF and EME are conditions with the JTF aiding the arm movement, while EMF and FME have JTF resisting the arm motion.

The torque slopes for each subject are shown in Fig. 3b. The torque slopes involving measurements that exceeded the hardware limit during the faster level (1 Hz) were calculated based on the neutral condition (NT) and slow level (0.5 Hz). For one subject that had both speed levels exceeding the hardware limit in FMF, the torque slope was computed assuming that the JND torque was 1.1 Nm for the slow level (0.5 Hz). To handle the outlier trials, analysis was conducted based on ranks: non-parametric tests were used, specifically the Kruskal-Wallis test followed by a Wilcoxon signed-rank test to find significant pairs. The Kruskal-Wallis test for the torque slopes in the dynamic condition yielded $X^{2}(3,~N=14) = 8.298, p = 0.040$. The significant pairs from the Wilcoxon pairwise comparison were FMF-EMF ($Z = 2.504, p = 0.012$) and FMF-EME ($Z = 2.228, p = 0.026$). These are indicated by asterisks in Fig. 3b.

**Table 3:** Medians and median torque slopes for the dynamic JND condition.

	FM2	FM1	NT	EM1	EM2
Ext. Torq. [Nm]	0.721	0.432	0.098	0.219	0.428
Ext. Torq. Slope [$\mathrm {Nm}/(^{\circ }/\mathrm {s})$]	2.625		$\cdot$	1.481	
Flx. Torq. [Nm]	0.799	0.430	0.101	0.314	0.408
Flx. Torq. Slope [$\mathrm {Nm}/(^{\circ }/\mathrm {s})$]	3.310		$\cdot$	1.409	

The first row (Ext. Torq.) shows the medians for the FME2, FME1, NTE, EME1, EME2 trials, while the third row (Flx. Torq.) shows the medians for the FMF2, FMF1, NTF, EMF1, EMF2 trials. The second row (Ext. Torq. Slope) shows the median torque slopes for FME, EME, and the fourth row (Flx. Torq. Slope) shows torque slopes for FMF, EMF. (Flx. = flexional; Ext. = extensional; Torq. = torque.).

A Kaiser–Meyer–Olkin (KMO) test found that the data is not adequate for factor analysis (KMO = 0.572) and a Bartlett’s test did not find statistical significance $X^{2}(3) = 6.272, p = 0.099$.

In the dynamic condition, all of the trials except the fastest ones (FM2 and EM2) had response times of $0.431 \pm 0.107$ s (very similar to the static conditions), while the fastest ones had response times of $0.244 \pm 0.064$ s. This difference was due to the experimental setup, where the subjects were asked to answer within the arm’s movement. With the faster arm motion, there was a shorter time window in which subjects could respond.

## Discussion

Overall, the elbow torques required to be noticeable (0.1–0.8 Nm under various conditions) are 100–200 times smaller than the maximum isometric torques able to be produced by the elbow (49–72 Nm)^12–14^. The results from this study show that the perception of torque feedback is affected by various joint conditions.

### Static conditions

The masking effect due to a static preload (Fig. 2a) resulted in a similar pattern with the previous test that was conducted with 0.64 Nm and 1.28 Nm^3^, but with smaller JND values in all condition levels as compared to the prior experiment. Feyzabadi et al. found a value of 0.012 Nm as the average of testing the neutral conditions. This is smaller than our results for NTE and NTF, which were 0.098 [0.061, 0.135] Nm and 0.097 [0.061, 0.132] Nm, respectively. Similarly to our previous test^3^, the JND of extension torques was larger when the subject was contracting their tricep (ECE) as compared to their bicep (FCE). It is unknown why this asymmetry occurs.

Additionally, the observed torque slope (JND torque divided by preload torque) in the current experiment was slightly smaller than that found in the prior experiment^3^. For the extension torque, it was 0.081 [0.050, 0.111] Nm/Nm for ECE (previously 0.118 Nm/Nm), and 0.011 [-0.020, 0.041] Nm/Nm for the FCE (previously 0.026 Nm/Nm). The flexional torque slope was 0.046 [0.015, 0.076] Nm/Nm for ECF (previously 0.0692 Nm/Nm) and 0.047 [0.016, 0.077] Nm/Nm for FCF (previously 0.0548 Nm/Nm). These differences are likely due to increased amounts of training in this experiment (we examined additional conditions, as compared to previously when only the static cases were tested) or due to slightly different parameters in the psychophysics tool that allowed the tests to converge faster. Additionally, in this experiment the direction that converged first could keep converging, whereas previously it stopped after a fixed number of transition points.

### Dynamic conditions

The dynamic conditions resulted in comparatively large values for JND torque (up to 1.1 Nm for some participants, with medians up to around 0.8 Nm for the fastest speeds), so motion does appear to cause a masking effect, confirming earlier studies^27,28^. For comparison, the maximum JND torque during the static conditions was only 0.2 Nm. Also, the dynamic conditions had a relatively large variance as compared to the static conditions (the largest observed dynamic JND torques were around 1 Nm more than the smallest dynamic JND torques). This may have been due to the relative difficulty of the experiment: participants needed to maintain a constant arm speed and track visual cues while responding to torque cues. Some participants may have been better able to focus on the torque cues while still maintaining the correct arm speed. The dynamic JND torques do increase roughly linearly with the arm speed. Thus, Weber’s law appears to be in effect, similar to previous results^1,25^.

The torques, and torque slopes, in the dynamic tests were similar for a given arm motion direction, regardless of the direction of applied torque (Fig. 2b). That is, if the elbow was moving in flexion, the minimum perceivable torques and torque slopes were very similar for a given speed regardless if the applied torque was pushing in flexion or extension; the same was true if the elbow was moving in extension. Also, the torque slopes and the JND torques at a given speed while the elbow was flexing were close to double those while the elbow was extending. Although not all pairs of fixed effect levels had significance, the FMF torque slope was larger than EME and EMF.

It is unclear why this asymmetry occurs. When the torques are applied, the arm is moving at nearly a constant velocity, and the velocity is the same in both directions. Thus, there is not a clear dominance of the flexors or extensors. It is possible that if the arm is flexing, then the biceps are more strongly contracted and if the arm is extending, then the triceps are more strongly contracted. However, this would result in an opposite pattern to that observed with the static conditions (FCE vs. ECE), where the bicep contracting had a smaller JND torque than when the tricep was contracting.

While the flexors tend to create higher MVC forces^32,33^, the extensors have a larger cross-sectional area^32^. A larger cross-sectional area of the extensor with the corresponding larger MS population might be responsible for the lower medians in extensional motion.

Another possible explanation for the difference between flexion and extension JND torques is related to the arm’s passive stiffness. The arm’s passive stiffness is slightly higher when the arm is flexed as compared to when it is extended^34,35^. Thus, if the exoskeleton causes the arm to move, a higher muscle stiffness will correspond to a smaller displacement. If the MS in the arm muscles sense the arm’s displacement, and contribute to the sensation of external torques, presumably individuals would have a harder time sensing smaller displacements. This corresponds to FMF having a higher JND torque slope than the EME condition (Fig. 3b).

A final possibility is that the pressure around the arm from the exoskeleton cuff might affect the results during elbow flexion. To mask the skin pressure, we applied distributed pressure around the arm. This pressure increased during arm flexion due to the expanded volume of the contracted bicep muscle (Fig. 1). Though we did not measure how much the FSR readings increased during full arm flexion, the escalation of the skin pressure might mask proprioceptive sensations even more, leading to the FME and FMF conditions being relatively insensitive.

The change rate of the perceived torque as a function of speed is much steeper than results from Zadeh et al.^25^. They used a Phantom haptic device that the user held in the fingers and moved back and forth laterally while a force either opposed or aided their motion (the force switched direction as the hand switched direction to maintain opposing or aiding). Zadeh et al.’s test yielded a JND of 0.222 N/(m/s) for forces opposed to the movement, and 0.187 N/(m/s) for aiding forces. To compare our results, we can convert our torque and speed based on an average length between the elbow joint to the center of the hand. Considering this moment arm to be 32.7 cm according to anthropometric data^36^ results in torque slopes of 0.884 N/(m/s) for EME, 1.668 N/(m/s) for FME, 1.871 N/(m/s) for FMF, and 0.824 N/(m/s) for EMF. In the opposing direction (FME and EMF in our experiment), the smallest JND is 0.824 N/(m/s) (EMF), and the smallest JND in the aiding direction (FMF and EME) is 0.884 N/(m/s) (EME). Thus, our experiment found values that are around four times larger than those in the Zadeh et al. experiment.

These differences could be due to several factors. Since the Zadeh et al. experiment used a Phantom haptic device held in the fingers, there are many more proprioceptors that could have been stimulated. Not only could mechanotactile and vibrotactile sensors on the fingertips be used to sense forces, but other proprioceptors along the wrist and hand could have provided additional information. Second, the input feedback was triggered when the averaged arm speed (over the entire back-and-forth cycle or multiple cycles) was at certain values. The force reversed directions at the ends of travel, and it is possible that the user could have sensed those transitions in some circumstances. Finally, the speed level investigated in the experiment was much slower than our experiment. The speed levels we chose are nearly two and four times larger than their experiment’s high velocity (0.22–0.28 m/s). For both Zadeh et al.’s experiment and our test, slower speeds yield better perceptual resolution. Our higher arm speeds may have led to larger torque slopes through some nonlinear mechanism.

### Static versus dynamic conditions

Comparing the JND values for the static versus dynamic conditions, the converged torque values under the dynamic conditions (medians ranging from 0.219 to 0.799 Nm) were substantially larger than the values with the static conditions (0.097–0.197 Nm, including the neutral conditions) (see Fig. 2; Tables 2 and 3).

Recall that GTO are traditionally considered to measure muscle force while MS measure position and velocity of a joint^6^. We thus consider which sensors are being used to detect the joint torques in this experiment. One possibility is that the GTO are being used to detect the force change in the muscles. In most of the static conditions, the muscles are flexed due to the preloads on the arm. Since GTO measure the muscle tension, and the muscle tension changes slightly due to the torque from the exoskeleton, the GTO could be detecting the exoskeleton’s effects. However, we note that in the dynamic conditions, the thresholds for noticing the applied torque were much higher than the thresholds for the static conditions (more than four times higher for fast velocities). We then must consider the amount of muscle activity in the biceps and triceps during the dynamic conditions. At the ends of the joint travel, there will be substantial muscle activity due to accelerating and decelerating the arm’s inertia. However, in the center of the range of travel, the arm is moving at approximately steady state (Fig. 6b). While we did not measure muscle activity during the experiment, we can roughly surmise the muscle activity in the biceps and triceps. By taking the second derivative of the measured elbow angle and assuming a nominal moment of inertia for the forearm and hand, we find that the elbow torques even in the center of the range of motion are frequently 1-3 Nm, which must be created by the biceps and triceps muscles. Additionally, the muscles surely have some co-contraction due to needing to maintain control and follow the desired trajectory. Thus, there can be muscle contraction in the dynamic conditions that is even larger than that tested in the static conditions. Even if the GTO were the sole arm sensor being used to measure the torque pulses from the exoskeleton, and the GTO are not sensitive to arm motion (as is commonly understood), then the additional muscle activation in the arm could account for much of the difference between the static and dynamic conditions.

An alternative hypothesis is that some combination of the MS and GTO are contributing to the sensation of torque from the exoskeleton. Since the MS primarily respond to length and velocity of the muscle fibers, they could be stimulated if the exoskeleton caused small changes in the arm’s position which were then detected and used to determine that the exoskeleton caused torque on the arm. One study^37^ found that people could correctly sense the direction of motion if their elbow was displaced only $0.1^\circ$–$0.2^\circ$ at angular velocities of greater than $1^\circ /$s. In our experiment, during the static condition torque pulses the exoskeleton itself moved at a maximum speed of 1–$2^\circ /$s with displacements of 1–$2^\circ$ during many of the pulses. While the exoskeleton’s motion is not exactly the same as the arm’s motion due to the intervening padding, it is likely that the arm moved more than 0.1$^\circ$–0.2$^\circ$ and thus could be triggering the MS with the exoskeleton’s torque pulses. During the dynamic conditions, the arm was moving at close to $97^\circ /$s or $213^\circ /$s in the different conditions. Due to the arm’s motion, it is extremely difficult to determine if any additional motion was caused by the exoskeleton. However, since the torques applied during the dynamic conditions were generally larger than those applied during the static conditions, it is likely that some perceptible arm motion occurred as well.

If the MS are contributing to the sensation of torque, then their contribution could be affected by the arm’s velocity. Assuming that Weber’s law holds, neural signals due to the arm’s motion could be thus masking the additional length changes due to the exoskeleton torque. Indeed, a recent model of the arm’s proprioceptive abilities supposes that the MS and GTO combine their signals^38^, and that the overall neural stimulus is a combination of the force and velocity.

An alternate reason why the dynamic JND is much larger than the static JND could be thixotropy^6^. Briefly, the response of the MS is affected by the muscle’s activation immediately preceding a test condition. A conditioning contraction will increase the background firing rate of the MS^39,40^, which would imply that a larger stimulus would be required to produce a noticable response. In the dynamic tests, the arm muscles create large torques (up to 8-10 Nm in the faster condition) when reversing the arm’s direction of motion. These large torques could mask any sensations in the center of the arm’s range of motion where the torques were lower, if the MS were involved in sensing the exoskeleton torque.

A final reason for the difference between the dynamic and static conditions could be possible psychological distractions during the test itself. The static condition experiment was comparatively much simpler than the dynamic condition, where people needed to first maintain a steady arm motion and then secondly detect disturbance torques. Thus, a larger stimulus might be needed to gain their notice. However, this is unlikely to explain all of the differences observed.

### Limitations

We have quantified the perceptual ability of the elbow to sense torques via a psychophysics tool. However, the accuracy of the converged values could be improved. The step size we implemented has a limitation as it is pre-defined as the exponential function. If subjects missed initial trials, it took a long time for the torque magnitude to get back to the perceivable range, which decreased resolution. The step difference could be adjusted to an individual’s learning speed using various adaptive algorithms in the future. Moreover, measuring electromyography (EMG) concurrently with torque inputs could better compare the arm’s muscle activity between dynamic and static conditions. EMG sensors could clearly show the muscle activity during the dynamic conditions, and better be able to determine if the increased JND values were due solely to increased muscle activity or if the MS must be involved. Furthermore, choosing subject groups with different age groups or strength-training proficiency will be another helpful reference for simulating real-life training scenarios because they have different muscle properties and proprioceptors. Younger people tend to have stronger MVC and larger cross-sectional muscle area^32^, and may have different proprioceptive abilities^6^.

Also, we did not perform any test re-test (i.e. using the same subjects on different days) to test the stability of the measurements or if subjects improved with additional practice. Thus, the stability of these measures are unknown. A final limitation is that suppressing the tactile feedback over the forearm and upper arm area potentially affected the measurements. The exoskeleton cuffs also intensified the skin pressure during the arm flexion due to the expanded volume of the flexor muscle groups, which could have further affected the measurements in unknown ways. In most real-world systems, exoskeleton cuffs will not be so tight and so the minimum perceptible torques may be different than those presented here.

## Conclusions

In conclusion, this work provides a comprehensive examination of the minimum elbow torques required to be sensed under various conditions with the GTO and MS in the arm. The analysis suggests that the perception of elbow JTF is influenced by the arm’s contraction condition and movement, and that these proprioceptors are less sensitive than the sensors in the skin. In general, we observed a more significant masking effect from arm movement than from isometric contraction. This work is useful for motion training systems, exoskeleton design, and force-feedback systems.

## Methods

### Elbow exoskeleton

**Figure 4:**
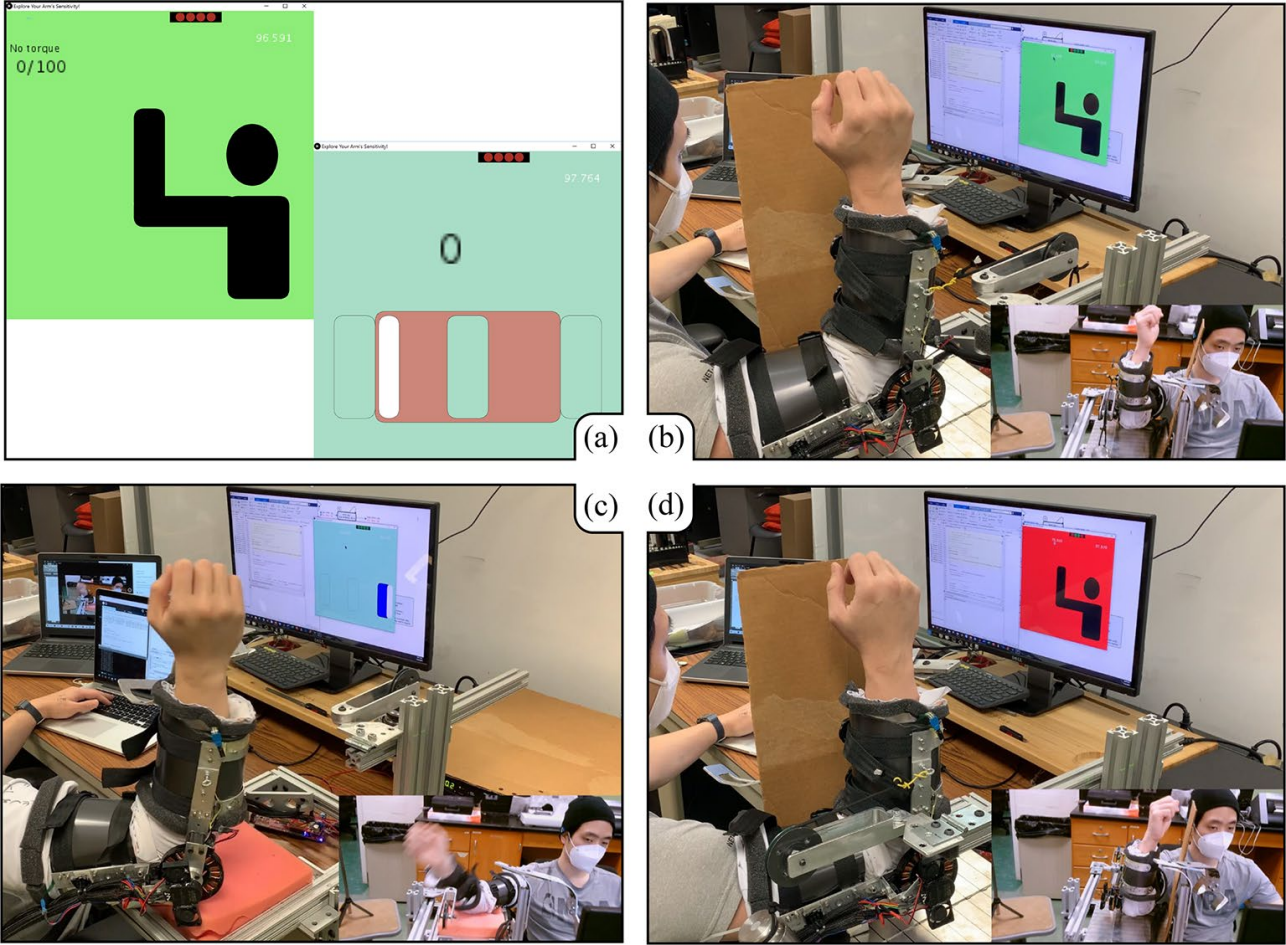
(**a**) GUIs for the static (left) and dynamic (right) mini-games. The score for the mini-game provides participants whether the current user’s response is correct or not. Additionally, the red block of the dynamic condition is only shown during the demonstration to supplement the ideal timing window for response. The white box represents the arm position that moves along three boxes that correspond the arm’s range of motion. From (**b**) to (**d**) are examples of testing of the static (flexor muscle contracted), dynamic, and static (extensor muscle contracted) conditions, respectively.

The hardware setup was an evolution of that used in a prior study^3,4^, and is shown in Fig. 4. The exoskeleton’s range of motion is mechanically designed to reach 120$^{\circ }$ when the arm is fully flexed. However, when it is worn with the foam, the range is reduced to 90$^\circ$–95$^{\circ }$ depending on the subject’s arm volume. The chosen motor was Antigravity MN7005 KV115 (24N 28P). To provide more torque to the arm for the dynamic condition experiment, the maximum current magnitude was increased to 14 A. The torque in the system was calibrated using a FUTEK LBB200 load cell with a Tacuna Systems Amplifier v.2.3. The torque constant was found to be 0.072 Nm/A.

As briefly described in the prior study^3^, we calculated how much pressure was required to mask the skin receptors based on measurements^26^. Tan et al.^26^ explained that the human tactile system is sensitive to the perimeter of the contact between the arm and another object. The JND force threshold per contact perimeter was found to be 0.06–0.09 N/cm^26^. The effective surfaces of the exoskeleton are the two plastic cuffs (Fig. 1b), of which the outer edge is 34.5 cm for each side and the surface area is $414~\mathrm {cm}^2$. When choosing 0.09 N/cm for the force threshold at the perimeter, the minimum pressure to mask the tactile sensors becomes 0.015 $\mathrm {N/cm}^2$. After donning the exoskeleton and tightening the straps, four FSR sensors (size: 4.5 by 4.5 cm) are measured to make sure that each sensor reading is more than $0.015~\mathrm {N/cm}^2$. The four FSR sensor readings are shown in the top right corner of the Graphical User Interface (GUI) as four red circles (Fig. 4a). Because we wrapped the arm with foam, the actual pressure required to be noticeable is assumed to be larger than the value calculated from the periphery.

### Software systems

The experiment was programmed in three different platforms: Processing, Texas Instruments Code Composer Studio (CCS), and MATLAB. Processing was utilized to make a Graphical User Interface (GUI) to give cues to the participants (Fig. 4); the subjects responded to perceived joint torques through a computer keyboard. Processing was also used to take the user’s responses and calculate the magnitude of the upcoming applied torque via a psychophysics tool. The calculated torque was then transmitted back to a Texas Instrument(TI) C2000 TMS320F28069M microcontroller (programmed in CCS) that commanded the TI DRV8305 motor shield to apply the torque to the arm. When the current was applied, to mitigate abrupt current surges that might trigger the wearer’s vibrotactile sensation, we apply a first-order Low Pass Filter with a rise time of 0.5 seconds. Finally, MATLAB took the logged data from Processing and summarized the user’s perceptual abilities. The microcontroller transmitted 24 byte data packages at 200 Hz, while the UI platform logged the data at 200 Hz and refreshed the GUI at 100 Hz.

#### Psychophysics tools

**Figure 5:**
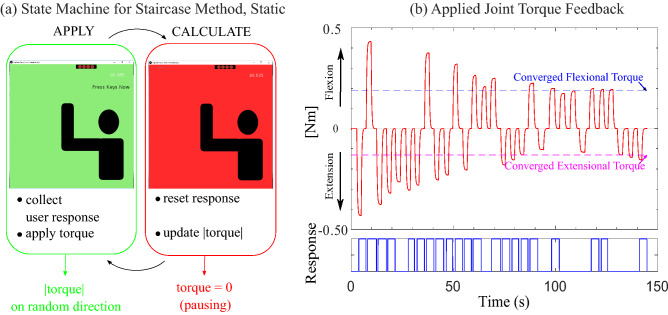
(**a**) Schematic diagram of state machine for static condition, and (**b**) example of a static JND test result. The programmed state machine (**a**) switches state between APPLY (applying torque) to CALCULATE (pausing torque). The APPLY state determines the direction of the torque randomly. The applied torque is graphed in the top panel of (**b**). Flexional torque is shown on the positive *y*-axis, and extensional torque is plotted on the negative *y*-axis. The CALCULATE state updates the magnitude of the torque based on the user’s response. The user responses versus time are shown in the bottom panel of (**b**), where a high value represents “perceived,” and a low value represents “not perceived.” A series of “perceived” responses leads to a decreasing input magnitude while the consecutive “not perceived” answers lead to the torque increasing. This continues for a specified number of transition points. Both directional torques converge to final values that are illustrated in the top panel of (**b**) as a blue dashed line for flexion and a magenta dashed line for extension.

A psychophysics tool similar to that used in Kim et al.^3^, the interweaving Staircase Method, was used to assess perception capability for both static and dynamic conditions. The Staircase Method is a recurring algorithm that updates the stimulus reflecting the user’s response (Fig. 5). It updates like a staircase; while the input alternates its direction randomly, the consecutive magnitude of the input increases or decreases based on the user’s response^1,25^.

In the Static experiment, the state machine (Fig. 5a) constitutes two states: the Apply state and the Calculate state. In the Apply state, the torque is provided to the user’s arm, and they make a response. The torque is set to zero in the Calculate state, and the state machine is updated to prepare for the next torque application. When torque is applied, the user’s response could be: (1) torque felt in the flexional direction, (2) torque felt in the extensional direction, or (3) did not feel any torque. A series of answers of “YES-torque felt” are responsible for decreasing the magnitude until the answer switches to “NO-did not feel torque” and vice versa. Whenever the trend of magnitude change switches from increasing to decreasing and vice versa, the transition is counted, and the step size of the magnitude is reduced to yield a high-resolution converging value. The step difference is calculated with the exponential decay $StepSize[n] = A e^{-0.1(n-1)}$, where *n* is the transition point number (starting at $n=1$), and *A* is a fixed scalar of $A = 0.1$ Nm for all conditions. The exponential function is tuned such that the initial adjustment can be done with the large step size and start decreasing as the transition happens. Thus, the Step Difference does not change until the transition happens. For example, when there were a series of YES responses, the system subtracted the same magnitude of Step Difference from the original torque value until the subject answered NO. When the answer switches from YES to NO, then the step difference’s size was changed. However, the initial magnitude of the torque is set considering the difficulty of the task. We found that the subjects had difficulty responding whenever the required arm movement is the same as the direction of applied torque from the pilot test. Therefore, the initial values for those conditions (FMF, EME) were set to 0.9 Nm, while other dynamic conditions have 0.5 Nm. The initial values for the static condition were 0.4 Nm. Even though more transition points directly contribute to a higher resolution of the converged value, the experiment needs to balance the resolution and fatigue from wearing the exoskeleton. Therefore, the static experiment continued until both the flexion and extension directions had at least eight transitions. Typically one of the directions converged before the other; the direction that converged first continued updating until the other direction reached eight transitions. At the end of the test, the mean of the final two transition points was recorded as the converged value. In the Pausing state, the experiment could also be stopped by the experimenter for an extended period of time. This was used whenever the participant’s arm strayed away from the initial neutral position too much (i.e., the forearm was more than around 30$^\circ$ away from vertical). If the experiment was stopped, the experimenter told the participant how to move their arm, so it was once again close to the neutral position, and then the experiment was resumed. The effect levels for the static condition are slightly different from our prior test^3^, specifically the smaller amount of preload was increased from 0.64 to 0.89 Nm. The design change was made to anticipate more significant changes in user response with the higher preload.

#### Psychophysics tool for dynamic JND testing

The psychophysics tool for dynamic testing shares many properties with the staircase method with the static condition, including the same exponential function to calculate the step difference. However, the dynamic JND experiment required additional design considerations because the participants need to maintain the required arm speed while reacting to the psychophysical input. The first design consideration was how to discretize the arm movement. We pilot tested applying torque during the whole arm cycle, as did prior researchers^25^; in this case, the subjects were asked to move the arms back and forth with the same average speed, and the torque magnitude and direction did not change during one complete cycle of extension and flexion. However, we noticed that users’ answers were mainly acquired when their arms were changing direction. At these moments, the velocity was very slow and not distinctly in either flexion or extension. We speculated that the users were feeling the torque in a condition that was essentially static. So, the final system only applied torque in the center of each arm’s movement while the arm had substantial velocity.

**Figure 6:**
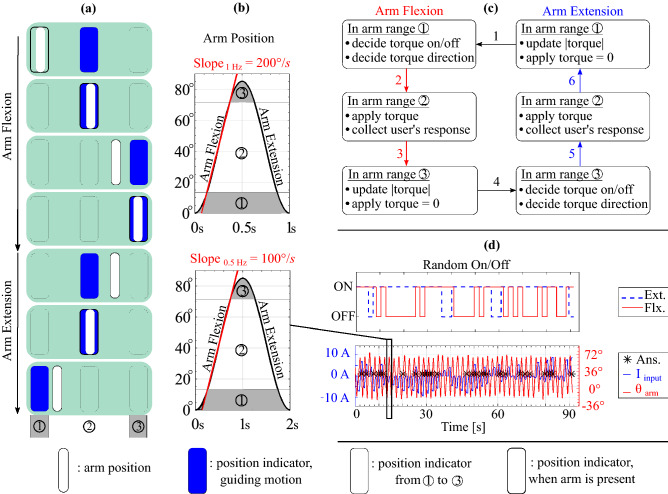
(**a**) GUI system guiding the arm movement. Participants are instructed to move their arm (white bar) to match the guiding block (blue block). The horizontal position of the arm position corresponds to the actual elbow joint angle. (**b**) Illustration of the commanded arm angular position versus time for the fast (1 Hz, top) and slow (0.5 Hz, bottom) velocities. The average speed over the center of the arm position range is shown with the red line. The gray shaded ranges of arm position correspond to the portions where the arm is changing speed ($\textcircled {1}$ and  $\textcircled {3}$); the white regions are where the arm was moving at roughly a constant speed, and torque was applied ($\textcircled {2}$). (**c**) State machine illustrating how torque is applied to the arm depending on the participant’s current arm position and if their arm is moving in flexion or extension. State transitions 1 and 4 occur when the arm changes its direction of motion; the other state transitions occur based on the arm position. (**d**) Example results from a trial. The top graph illustrates how the random on/off works on each extensional/flexional torque input. The bottom graph shows the subject moving their arm back and forth (red line) and the subject reacting to the current input (blue line). The black asterisks show the moments when the subject responded.

Figure 6 gives an overview of the GUI, arm motion, state machine, and example data for the dynamic conditions. To guide the participants’ motion, a GUI was presented on a monitor. Figure 6a shows a series of green rectangles which are examples of the GUI at different points in the arm’s motion. Within each green rectangle, there are three blocks (outlines of rectangles) that correspond to the limits of the arm’s range of motion and the center of the range of motion. These are labeled $\textcircled {1}$ - $\textcircled {3}$ at the very bottom of the sequence of green rectangles. Each block changes its color to blue sequentially to guide the wearer to move to that arm angle. A moving white bar displays the wearer’s current arm position. The border of each block becomes thicker when the arm position hits the block, indicating the arm reached different points in the range of motion. Additionally, to cue the user, the moving white block turns red when the subject presses the keyboard. This process ensured that the subjects moved through the full range of motion. The approximate arm angles swept by the participant are shown in Fig. 6b. The gray shaded regions and labels $\textcircled {1}$ - $\textcircled {3}$ correspond to the regions and labels indicated at the bottom of Fig. 6a.

The state machine, Fig. 6c, updates and generates the torque signal based on the participant’s past reply during one motion direction (i.e., flexion or extension). The state machine calculates the stimulus based on the real-time arm position. Whenever the subject’s arm moved the wrong way or missed the end block, the state machine detected it and paused the internal update such that the user’s response was not recorded and no input magnitude change was made. When subjects respond, because two options (Yes or No) are given instead of three (flexion, extension, No), the system is equipped with a random-on-off system to mitigate subjects guessing the incoming stimuli. Figure 6d shows how the random decision on each extension or flexion affects the torque generation.

The second design problem in the dynamic conditions was the means to effectively guide the participant into moving their forearm back and forth at the right velocity. In pilot testing, we tested solely using a visual cue to guide arm motion, similar to the study from^25^. However, the subjects were easily distracted by the arm motion directions and could not focus on the psychophysics sensations. Therefore, we used multimodal cues (an auditory cue in addition to the GUI) to facilitate subjects adapting to the test environment faster. We first attempted to use a continuous sound where the pitch was synchronized with the arm’s motion, but this was not helpful. Even with a small deviation from the desired movement, the sound changes were confusing to the subjects. We then sectioned the arm’s range of motion into five regions and assigned music notes of do-le-mi-fa-sol to each region using the Processing 3.0 Minim library. The programmed sound played whenever the arm position was within the region. Even with this system, the motion was not as smooth as expected. Eventually, we decided to use two different beeping sounds played whenever the arm motion should change direction. The subjects were instructed to move their arm to hit the end blocks (Fig. 6a) concurrently with the beeping sounds. This synchronization prevented the torque from being applied during the wrong arm movement.

Comparing Fig. 6b, d, the users nominally would move their arm through a range of $90^{\circ }$. In practice, people changed their arm’s motion direction as soon as they hit the end blocks ($\textcircled {1}$ and $\textcircled {3}$), without fully flexing or extending their arm. This led to a slightly reduced range of motion during the experiment.

### Participants

We fully randomized the session orders for the static and dynamic conditions via a Standard Latin-square design to account for any fatigue effects and learning effects. The recruited sample size was $N = 14$ where 12 were male, 2 were female. The average age was $29.31 \pm 4.00$ years, and all were healthy right-handed individuals. The experiment was approved by the Virginia Tech Institutional Review Board (IRB #16-175) and the Human Research Protection Program at Virginia Tech for the COVID-19 mitigation process, and all experiments were performed in accordance with the relevant guidelines and regulations. All the participants also followed the COVID-19 safety guidelines. Subjects provided informed consent and signed a consent form, then were instructed about the purpose of the experiment before the test. The entire experiment took 45-50 min, including a 5-min break between the two sessions.

### Experimental protocol

All of the experiments began with calibration, discussed in the next section. The tests constituted a randomized combination of static and dynamic sessions. The static and dynamic sessions were made up of a practice session, followed by two test sessions with different effect levels. Thus, all the test sessions, including static and dynamic tests and fixed effect levels, were fully randomized. The practice session was intended to facilitate learning how to respond with the keyboard given a JTF input. Due to the difficulty of the experiment setup for the dynamic conditions, a practice session was conducted whenever the effect level changed. For instance, when the speed level was increased, another practice session was conducted immediately before the test. The practice session for the static condition was conducted only at the neutral arm posture, with no preload torques.

All the practice sessions were comprised of a mini-game. Participants were rewarded with 10 points for a correct answer and punished with -5 points for a wrong answer. Figure 4a illustrates the GUI for the static (left) and the dynamic (right) mini-games. Upon responding, the correct answer is shown on the GUI by updating the score. Subjects are required to score 100 to transition into the test sessions. The mini-game that took 3–4 min in our previous work^3^ was extended to be 10 min long to ensure each participant got used to the system. After each session, to promote engagement, a brief summary of the participant’s performance was provided to them. The provided analysis included the smallest magnitude the subject could perceive in each direction and how quickly they responded.

Throughout the test, two computer monitors were used for the experiment: one for the subject and another for the experimenter. Thus, the experimenter observed the subject’s converging dependency to determine if the subjects were correctly pressing the buttons or were distracted from the environment. If a subject was distracted, the experiment was paused and the experimenter reminded them to pay attention.

#### Calibration

Subjects first adjusted the seat and height of the platform where their elbow was resting (labeled “stand” in Fig. 1b) until their upper arm was parallel to the ground. The exoskeleton was adjusted so the motor was aligned with the center of rotation of the elbow joint. Next, calibration of the strap pressure was conducted in the arm’s neutral position (at a right angle), to account for the expanded volume of the arm flexors. During the donning process, the subject’s arm was kept fully supinated. Straps were fastened until four FSR readings reached the minimum required values, which were indicated on the GUI as red circles at the top of the display (Fig. 4a). Due to the relatively high pressures from the exoskeleton cuffs, subjects were asked to provide feedback on whether it was too snug while the system was being demonstrated, and the strap pressure was adjusted accordingly. Lastly, subjects finalized the calibration process by resetting the index pin. As soon as the encoder detects the index pin during the arm swing, it triggers the timer interrupts in the microprocessor and prepares the motor controller.

Once the calibration is finished, the GUI indicator directs the instruction to conduct the Interweaving Staircase Method. The state machine indicates when each experiment terminates, then resumes once the conditions for the next experiment are ready, such as changing the weight used to create the preload torque in the static conditions.

#### JND for static condition

The preload torques were applied by a string pulling on the forearm of the exoskeleton. The string passed over a pulley, and a mass of 700 g or 1000 g of mass was attached to the string on the far side of the pulley (Fig. 4b, d). This created a constant preload torques of 0.893 Nm and 1.275 Nm due to the string pulling on the exoskeleton forearm at a distance of 13 cm from the elbow joint. On top of these preload torques, the exoskeleton motor provided test torques.

The GUI notifies the subject that a torque event occurs via a color change, the only visual data transferred. The green background on the GUI (Fig. 4b) means the torque is being applied, and the subject should respond if they felt the torque feedback. The red background on the GUI (Fig. 4d) indicates that the torque is not applied and the subject should prepare for the upcoming torque input. While the red background is shown, any user response is not recorded. From pilot testing, we found that the recovery torque (i.e., torque returning to zero after a test stimulus is applied) behaves as a phantom stimulus in the opposite direction. The event cue (color change only when the stimulus is applied) keeps subjects from reacting to the restoring torque.

A panel obscured the exoskeleton from the participants’ view to prevent them from responding by watching the arm movement (Fig. 4b, d). The participants were instructed to not touch the panel, as the mechanoreceptors around fingers might respond more sensitively than the kinesthetic receptors. This better matches a typical motion training scenario that assumes a floating limb while correcting the motion. Test subjects are required to relax their right arms in an L-shape, not fully supinated nor pronated. Subjects cannot see their arm, so they can easily deviate from the neutral position when torques are applied. If this occurs, the experimenter pauses the state machine and provides verbal correction to be in the neutral position again.

#### JND for dynamic joint condition

The dynamic condition sessions required knowledge that could be difficult to explain by simple verbal explanation. Therefore, a pre-recorded video of the instructor reacting to the GUI with the exoskeleton was prepared. It contained what the GUI was showing, the expected response, and what feedback the system would give when the answers were given to the system. Additionally, at the beginning of each practice session, the subjects were asked to move their arm while wearing the exoskeleton but without torque feedback. Torque was applied only after they had adapted to the visual and auditory interfaces and were able to generate the expected speed.

The test was separated into two sessions: torque in the opposing direction and torque in the aiding direction. When aiding, the torque was applied in the same direction as the arm’s motion, and when opposing it was in the opposite direction. Consequently, the flexional torque of FMF and extensional torque of EME were grouped together as the aiding trials, while EMF and FME were applied as part of the same session as the opposing trials. The expected user response was either yes or no (felt or did not feel the torque), and each direction’s torque was converged based on its own transition counter.

The dynamic condition had ten transition points to convergence. For the practice session of the dynamic test, we increased the number of transition points to fifteen so that the subjects could adapt to the exoskeleton and the GUI system before the test sessions. All the test combinations, including the static and dynamic, were fully randomized; some subjects had harder tasks to begin with than others. Specifically, those who began with the dynamic aiding conditions required additional time to learn the system.

## Supplementary Information

Supplementary Table 1.

Supplementary Information.

## Data Availability

The raw and processed data generated and analyzed during this study are included as Supplementary Information.
